# Clinical value of ambulatory blood pressure in pediatric patients after renal transplantation

**DOI:** 10.1007/s00467-017-3781-6

**Published:** 2017-08-25

**Authors:** Rafael T. Krmar, Jorge R. Ferraris

**Affiliations:** 1 Department of Physiology and Pharmacology (FYFA), Karolinska Institute, C3, Nanna Svartz Väg 2, 171 77 Stockholm, Sweden; 2 Departamento de Pediatría, Hospital Italiano de Buenos Aires, Juan D. Perón 4190, C1199ABB C.A.B.A, Código Argentina

**Keywords:** Renal transplantation, Children, Hypertension, Ambulatory blood pressure monitoring

## Abstract

Hypertension is a highly prevalent co-morbidity in pediatric kidney transplant recipients. Undertreated hypertension is associated with cardiovascular complications and negatively impacts renal graft survival. Thus, the accurate measurement of blood pressure is of the utmost importance for the correct diagnosis and subsequent management of post-renal transplant hypertension. Data derived from the general population, and to a lesser extent from the pediatric population, indicates that ambulatory blood pressure monitoring (ABPM) is superior to blood pressure measurements taken in the clinical setting for the evaluation of true mean blood pressure, identification of patients requiring antihypertensive treatment, and in the prediction of cardiovascular outcome. This Educational Review will discuss the clinical value of ABPM in the identification of individual blood pressure phenotypes, i.e., normotension, new-onset hypertension, white-coat hypertension, masked hypertension, controlled blood pressure, and undertreated/uncontrolled hypertension in pediatric kidney transplant recipients. Finally, we examine the utility of performing repeated ABPM for treatment monitoring of post-renal transplant hypertension and on surrogate markers related to relevant clinical cardiovascular outcomes. Taken together, our review highlights the clinical value of the routine use of ABPM as a tool for identifying and monitoring hypertension in pediatric kidney transplant recipients.

## Introduction

Hypertension is a central risk factor for the development of cardiovascular disease and a major cause of morbidity and mortality in the general adult population [1–3]. Hypertensive children with underlying chronic kidney disease are at increased risk for cardiovascular disorders [4, 5]. Hypertension is also a highly prevalent co-morbidity following renal transplantation in children and is associated with hypertension related cardiovascular complications [6]. Since hypertension is a modifiable risk factor, early recognition and intervention can substantially decrease hypertension associated morbidity [7–9].

In recent years, there has been renewed interest in the field of pediatric hypertension and post-renal transplant hypertension. This has been reflected by the publication of international guidelines for the optimal diagnosis and management of hypertension in children and by a growing number of review articles on the topic of childhood hypertension post-renal transplantation [10–19].

In the adult population, the introduction of ambulatory blood pressure monitoring (ABPM) has changed the way we look at blood pressure [20]. In sharp contrast to blood pressure readings taken in the clinical setting, ABPM is much more suitable and robust in identifying those patients requiring antihypertensive treatment [21, 22].

Although the use of ABPM in the workup of children with hypertension has gained increasing attention [17, 23–27], there have been limited data available on the routine use of ABPM following renal transplantation [28].

This Educational Review will consist of an examination of contemporary clinical studies on the assessment of blood pressure in pediatric kidney transplant recipients. The main objective is to highlight the clinical value of ABPM after renal transplantation, thus enhancing the accuracy of blood pressure readings and facilitating clinical decision-making. Although our review is primarily based on cross-sectional studies, it also focuses on published longitudinal studies applying ABPM. Specific areas such as the pathophysiology of post-renal transplant hypertension and the treatment of pediatric hypertensive kidney transplant recipients will not be covered here since these topics have been extensively discussed in recently published review articles [13, 15, 18, 19]. Although references have been selective, our review is intended to provide health professional with a concise, clear, and independent source of information.

Finally, we highlight the disadvantages of ABPM, including limited availability at certain centers and the impracticability of ABPM for patients required to travel long distances. We thus conclude that rigorous blood pressure measurements taken in the clinical setting are still useful provided that they are strictly performed according to validated protocols [12, 29–31]. In particular, it is worth pointing out that the oscillometric method of measuring blood pressure in the clinical setting is increasingly being used due in part to the widespread implementation of policies banning the use of the mercury sphygmomanometer [32, 33]. Current international guidelines still recommend the use of the auscultatory method to confirm hypertension detected by the oscillometric method [12, 29]. There is also evidence derived from adult studies on the clinical usefulness of home blood pressure monitoring [34, 35]. In children, home blood pressure monitoring has also been recognized as a reliable tool for the assessment of blood pressure, albeit not extensively used [12]. Of note, in the clinical setting, where physicians face mounting demands on their time, the adherence to current recommendations becomes problematic [32, 33]. Having this in mind, we have recently constructed oscillometric blood pressure tables to facilitate health provider’s interpretation of oscillometric clinical blood pressure readings [36].

## Blood pressure measurement in the clinical setting and outside of office visits: Are they comparable?

It should be recognized that blood pressure taken in the clinical setting is highly variable and influenced by several factors such as position of the arm and back, respiration, emotion, exercise, pain, disease, drugs as well as the circumstances of the measurement itself [37–40].

If blood pressure variability is ignored by the observer, then the risk of an erroneous diagnosis becomes high. The analysis conducted by Myers et al. showed that in adult blood pressure measurements obtained outside of the clinician’s office are systematically and significantly lower than blood pressure readings obtained in the clinical setting [41]. In order to obtain a better estimation of the subject’s actual underlying blood pressure, pediatric and adults guidelines acknowledge that the diagnosis of hypertension in the clinical setting should be based on multiple blood readings taken on several separate occasions [1, 3, 12, 29]. Accuracy in measuring blood pressure is therefore of the upmost importance in the correct diagnosis and proper management of hypertension. Additionally, blood pressure taken in the clinical setting is prone to clinical inertia, i.e., failure of health care providers to initiate or intensify therapy when indicated, which ultimately jeopardizes the proficiency of care provided [42].

Having briefly examined the inherent limitations of measuring blood pressure in the clinical setting, it becomes apparent that less biased and more accurate methods of representing patient’s actual blood pressure patterns are needed.

The technique of non-invasive ABPM was initially conceived for evaluating antihypertensive drug efficacy [43]. An important finding derived from early studies conducted in adults was the observation of discrepancies between clinical blood pressure measurements and ABPM in a group of treated hypertensive patients [44, 45]. This observation was of clinical significance since it raised the question of whether the inconsistencies between the methods would translate into a different prediction of risk. The publication of the seminal study by Perloff et al. showed the superiority of ABPM over blood pressure measurements taken in the clinical setting in predicting cardiovascular outcomes [46]. Subsequent studies confirmed that ABPM is a better predictor of risk than BP readings taken in the clinic setting [47–51]. Over the years, the prognostic superiority of ABPM has been demonstrated both across gender and ages as well as in treated and untreated hypertensive patients with different underlying disease states.

Home blood pressure monitoring also provides more useful information than blood pressure measured in the clinical setting [52]. However, a recent meta-analysis showed that home blood pressure measurements have less sensitivity when compared to ABPM as a single method for diagnosing hypertension in adults [53]. We recognize ourselves in having limited clinical experience with the routine use of home blood pressure monitoring in our renal transplant programs and that much of our knowledge is mostly derived from the literature. An alternative worth considering is that when there is a concordance between the ABPM and home blood pressure monitoring, the latter could be appropriate for follow-up of treated hypertensive pediatric patients [12].

Several pediatric studies sought to assess whether blood pressure readings taken in the clinical setting are sufficiently reliable when compared to ABPM in adequately categorizing a recipient’s blood pressure status after renal transplantation. Pooled data from four pediatric studies, including 188 untreated kidney transplant recipients, showed that the point estimate prevalence (adjusted Wald confidence interval) of recipients with normotension in the clinical setting, who in fact were diagnosed as having true or sustained hypertension by ABPM criteria, was 30% (95% confidence interval 24 to 37%) [54–57]. Also, the opposite condition, i.e., hypertension in the clinical setting while normotensive by ABPM criteria has been observed in untreated pediatric kidney transplant recipients (point estimate prevalence 9%, 95% confidence interval 5–13%) [54–57]. These figures are probably an underestimation of the magnitude of the problem. The observed wide confidence interval, which precludes estimating with precision the true prevalence of the variable of interest, is likely due in part to the sample size. Nevertheless, these two conditions, i.e., masked hypertension and white-coat hypertension, respectively, can only be diagnosed by means of ABPM [58–60]. Of note, masked hypertension and white-coat hypertension may ultimately impact the recipient’s health outcomes in a negative way if they go unrecognized. In children with chronic kidney disease, masked hypertension is associated with subclinical abnormalities in cardiac structure [61]. Importantly, both in adults and children, masked hypertension should be identified and treated adequately to control hypertension [62, 63]; whereas recipients with white-coat hypertension should not receive antihypertensive treatment [1, 12, 64]. Recipients with masked hypertension should be followed regularly with ABPM to guide the management of hypertension. Since children and adults with white-coat hypertension can progress to sustained hypertension [1, 65, 66], it is advisable to follow up with repeated ABPM to ensure timely intervention [1, 12, 66]. It should be stressed that if a patient’s blood pressure values obtained in the clinical setting are in the hypertensive range and in the presence of target organ involvement from hypertension, commencement of antihypertensive treatment should not be delayed. Since the chances of obtaining a normal ABPM in this particular clinical situation are low, it is reasonable that the use of ABPM as a diagnostic tool should be deferred and applied instead for monitoring the subsequent management of hypertension.

In treated hypertensive pediatric kidney transplant recipients, we and others have observed that readings taken in the clinical setting are not a solid surrogate for ABPM in identifying true responders to antihypertensive therapy. The point estimate prevalence of uncontrolled hypertension defined upon ABPM criteria in 457 treated hypertensive recipients was 54% (95% confidential interval 49–58%) [54–56, 67–75]. Here, we would like to indicate that the term undertreated hypertension should be used instead of uncontrolled hypertension since in our experience, as we discuss later, the routine use of ABPM has resulted in a high prevalence of blood pressure control, whereas resistant hypertension is a rather uncommon condition [76]. Attention should also be given to the fact that over the years, the recipients’ ambulatory blood pressure status has been defined in many different ways. Because of this, detailed conclusions are limited. The use of blood pressure loads, i.e., the percentage of blood pressure readings above pre-established cut-off values, as a diagnostic tool in the workup of pediatric hypertension might serve as an illustration. There is robust adult data arguing against the use of blood pressure load as a routine diagnostic tool [77, 78], and so far there is no conclusive pediatric research indicating major advantages to pediatricians in applying blood pressure loads for diagnosing hypertension or in evaluating antihypertensive efficacy. In adult studies, there is a high degree of consistency showing that mean ambulatory blood pressure values are suitable for the accurate diagnosis of hypertension and for the reliable prediction of hypertensive related organ damage [1]. In our view, the characterization of recipient’s ambulatory blood pressure status in a busy clinical environment should rely on definitions that are easy to deal with unless there are compelling reasons to do otherwise. In general, complexity resists simplification, and additional investigations in this area are certainly needed.

In this section, we have shown that the application of ABPM in pediatric kidney transplant recipients provides a better estimate of true or mean blood pressure level than blood pressure readings obtained in the clinical setting. Undoubtedly, all the above-mentioned studies provide supportive evidence for a real problem when attempting to define a recipient’s actual blood pressure status solely by means of blood pressure obtained in the clinical setting.

## Additional information provided by ABPM

Before going further, we need to consider what has been shown on the reproducibility of ABPM. It should also be acknowledged that ABPM devices have become more precise, with measurements now more reproducible than blood pressure taken in the clinical setting [79, 80]. Validation of ABPM for application to clinical practice in pediatric kidney transplant recipients requires comparison with established office blood pressure measurement techniques, so as to determine whether the two methods used to monitor blood pressure sufficiently agree in order to be used interchangeably. Bland and Altman proposed a method for assessing agreement between two methods of measurement, based on quantifying the variation in between-differences for individual patients [81]. Repeatability is relevant to the study of method comparison because poor repeatability, i.e., considerable variation in repeated measurements on the same subject, preclude the assessment of the amount of agreement that is possible. The examination of repeatability can be approached in the same way as the assessment of agreement [81]. We examined the degree of repeatability of office and ambulatory blood pressure measurements in pediatric kidney transplant recipients and observed that ABPM shows a better reproducibility than blood pressure recordings taken in the clinical setting [82]. Our observation extends previous results derived from adult studies conducted in normotensive and hypertensive subjects showing that standard deviations of the mean differences, which are used as a reciprocal of blood pressure repeatability, were lower for ABPM recordings compared to office blood pressure recordings [83–86]. In order to maximize the validity and reproducibility of ambulatory blood pressure values, a recent adult guideline suggests performing two consecutive 24-h periods [87]. Clearly, this is not to be considered a requirement in daily clinical practice.

There is a diurnal rhythm of blood pressure that mainly depends on the pattern of physical activity [88]. A normal decline of blood pressure during the sleep period ≥10% compared with daytime blood pressure is referred to as a dipper [1]. The normal diurnal rhythm of blood pressure has been observed to be absent in some hypertensive patients and this phenomenon has been shown to be associated with increased cardiovascular morbidity and mortality [50, 89–91]. In untreated hypertensive adults, both blunted nocturnal blood pressure decline and extreme dipping are associated with worse cardiovascular prognosis as compared to normal dippers [92].

Nocturnal blood pressure and circadian rhythm is often abnormal in adult and pediatric patients with chronic kidney disease as well as after renal transplantation [93]. This should carry the implication that ABPM would offer a unique perspective for chronotherapy, i.e., bedtime dosing of antihypertensive medication in patients displaying an abnormal non-dipping profile. In adults, the effect of chronotherapy remains to be elucidated [94]. In children, there are no data indicating benefit to the restoration of a normal circadian profile. In this regard, attention should also be given to the low reproducibility observed in children in the decline of blood pressure during the sleep period following renal transplantation [82].

There are, as described in the next sections, additional significant aspects from the clinical perspective.

## On the importance of the timely diagnosis of sustained hypertension and of achieving controlled blood pressure

Presently, the question of controlled blood pressure is of great importance since, as previously mentioned, many treated hypertensive patients have undertreated hypertension.

In our experience, controlled blood pressure in pediatric kidney transplant recipients was more than doubled when ABPM is performed yearly, thus conveying the message to pediatric healthcare providers and researchers of the usefulness of this technique [95]. Recently, our early results were confirmed by an independent research group that in a retrospective study evaluated ambulatory blood pressure phenotypes over time in 123 pediatric and young-adult kidney transplant recipients (76 recipients aged <18 years) who underwent at least two repeated ABPM (*n* = 98) [96]. Therefore, a paradigm shift is needed to account for these results since these data are consistent with evidence showing that the use of ABPM in the management of hypertensive adult patients is of clinical value [64].

Since high blood pressure is commonly asymptomatic unless the child has severe hypertension that is left untreated [97, 98], the use of biomarkers in place of a relevant clinical outcome to which blood pressure is correlated has become commonplace (e.g., blood pressure reduction in adults instead of stroke) [1]. Additionally, a surrogate marker may be more sensitive to drug-effect change obtained over a shorter time frame. In this section, we will focus our discussion on some renal and cardiovascular surrogate markers that are routinely used in the clinical management of childhood hypertension and those that have proven prognostic value in the adult population [1].

### Renal function

In adult patients with chronic kidney disease, controlled blood pressure slows the further loss of renal function [99]. Similarly, the results derived from the ESCAPE pediatric trial indicate that controlling blood pressure with a target 24-h mean arterial pressure below the 50th percentile significantly reduces the rate of progression to end-stage renal disease [100].

Although all transplant patients experience a decline in their renal function over time, adult studies have shown that for every 10 mmHg higher systolic blood pressure graft loss is increased by 12–15% [101, 102]. Consequently, the rationale to treat hypertension after renal transplantation is not only to prevent adverse cardiovascular outcomes but also to delay the progression of allograft loss. In a 2-year prospective interventional study aimed at improving blood pressure control by means of intensifying antihypertensive therapy and guided by applying repeated ABPM, Seeman et al. observed that in recipients with controlled blood pressure (*n* = 23), the estimated graft function remained stable whereas in recipients with uncontrolled hypertension (*n* = 8) allograft function decreased significantly over time [103].

More recently, Hamdani et al. observed that allograft function was significantly lower in recipients with sustained hypertension when compared to normotensive recipients in a large retrospective multicenter cross-sectional study including 221 participants that also used ABPM to assess blood pressure status and plasma creatinine to estimate renal function [54].

We explored the effect of hypertension, assessed by ABPM performed annually after transplantation, on the loss of renal function over a mean follow-up of 6.2 years by means of allograft function measurements, according to our local protocol, either by the renal clearance of inulin or iohexol [104]. Since the cohort study underwent ABPM at yearly intervals after transplantation, we assumed that the ABPM results obtained at each annual control would reflect the recipient’s previous year’s blood pressure status. We calculated the recipient’s post-transplant cumulative exposure to hypertension by summing the number of yearly periods of uncontrolled hypertension, including new onset hypertension. We observed that there was no significant difference in the effect on glomerular filtration slope between the hypertensive recipients (*n* = 44) and recipients that were normotensive or had controlled blood pressure throughout the entire post-transplant follow-up (*n* = 24) [104]. In the hypertensive group, the cumulative incidence of post-transplant uncontrolled hypertension represented 39% (95% confidence interval 31–47%) of their follow-up period. We infer that either the magnitude or duration of exposure to hypertension in our cohort study might have been insufficient for demonstrating a negative effect on recipients’ measured glomerular filtration rate or that antihypertensive treatment was indeed effective in slowing the impairment of renal allograft function [104].

### Left ventricular hypertrophy

In hypertension, left ventricular hypertrophy is the heart’s response to the presence of increased left ventricular load and neurohumoral stimuli, which results in an augmentation of oxygen consumption. In fact, myocardial ischemia is a hallmark of hypertensive heart disease [105]. As such, left ventricular hypertrophy is an ominous high-risk marker that demands urgent treatment. In adults, the introduction of effective long-term antihypertensive therapy resulted in a dramatic reduction in morbidity and mortality from hypertensive heart disease [106]. Left ventricular hypertrophy is also used as a surrogate outcome for cardiovascular risk in the pediatric population [12, 29]. In children with chronic kidney disease and left ventricular hypertrophy, previous controlled studies indicate that controlled blood pressure, defined as ambulatory blood pressure levels below the 95th percentile, was associated with left ventricular mass index regression and improvement of myocardial function [100, 107]. While such data is lacking in pediatric renal transplant recipients, the presence of left ventricular hypertrophy has been well documented in this high-risk population [54, 72]. In a recently published long-term consecutive case series study, including 68 recipients that were regularly followed with ABPM from the date of transplant surgery, we reported a low prevalence of left ventricular hypertrophy at last examination (7.6%, 95% confidence interval 2.5–17%) [104]. Although a weakness of this study was the retrospective design, the low prevalence of left ventricular hypertrophy might underlay the benefit from the systematic application of ABPM at yearly intervals for the evaluation and management of post-transplant hypertension [104]. Consequently, there is a reason to believe that controlled blood pressure after renal transplantation in recipients with left ventricular hypertrophy would be associated with a decreased left ventricular mass.

### Intima-media thickness

Since the introduction of the ultrasound-derived assessment of the combined intimal and media layers of the common carotid artery, the technique has gained acceptance as a marker of asymptomatic target organ damage and displayed prognostic value among subjects with hypertension [108, 109]. In the hypertensive patient, there is a positive association between systolic blood pressure and carotid intima-media thickness [110]. There is evidence of the prognostic value of carotid intima-media thickness to predict cardiovascular events both in the asymptomatic and in the diseased adults [111, 112]. The utility of measuring carotid intima-media thickness as a primary end-point in clinical trials where the objective is to evaluate the efficacy of antihypertensive therapy has also been extensively documented [113–115]. In children, the interpretation of carotid intima-media thickness measurements has largely been facilitated by the availability of normative reference values [116]. Also, it is likely that there is an independent association between blood pressure and carotid intima-media thickness in children [117].

Based on cross-sectional data derived from a large pediatric cohort including patients with advanced chronic kidney disease as well as renal transplant recipients, Litwin et al. speculated that successful renal transplantation could partially reverse the arteriopathy associated with chronic exposure to the uremic milieu [118]. Later, the same research group substantiated this proposition in a prospective study [119]. In a previous prospective study, we examined 22 recipients over 9 years with repeated echocardiography and carotid scans in a standardized manner after transplantation [120]. Also, in accordance with our post-transplant follow-up program, all participants underwent ABPM at yearly intervals after transplantation. In this study, we did not observe any statistical evidence of systemic changes in carotid intima-media thickness over time. Additionally, the prevalence of left ventricular hypertrophy at last follow-up was low (4.5%) [120]. Since we assumed a linear association between blood pressure and carotid intima-media thickness, we therefore infer that our results might reflect the effect of long-standing blood pressure control (point prevalence of controlled blood pressure at the last carotid scan was 82%, 95% confidence interval 56.5–96.2%) [120].

## Conclusions

Hypertension-related organ damage is a chronic process that takes years to manifest. Hence, reducing the cumulative burden of uncontrolled hypertension and cardiovascular injury should be a priority for clinical care and research in pediatric renal transplant recipients. Here, we emphasize that the routine use of ABPM offers advantages in terms of the accurate diagnoses of hypertension and refining risk-stratification when compared to traditional blood pressure measurements taken in the clinical setting. This makes ABPM an indispensable technique in the daily clinical practice for the management of hypertension following renal transplantation. In addition, economic factors also favor ABPM, for example up to 14% savings in the cost of healthcare provision has been calculated when ABPM is incorporated in the diagnosis and treatment process of hypertension in adults [121]. Pediatric studies also suggest that diagnostic ABPM in the initial evaluation of suspected childhood hypertension may be the most economically efficient diagnostic strategy [122, 123]. We have been performing ABPM and echocardiography at yearly intervals following renal transplantation since 1998, recognizing the fact that no studies have specifically addressed the appropriateness and cost-effectiveness of this approach [82, 95, 104].

In broad terms, our cut-off points to define ambulatory hypertension are based on percentiles derived from healthy children and adolescents rather than in relation to outcomes [124]. For many years, the use of a statistical instead of an operational definition has been recognized as an important limitation in defining childhood hypertension, and progression in this area has been slow [11, 12, 29]. Although it is unknown whether differing ambulatory cut-off points below the 95th percentile would reduce post-transplant hypertensive end-organ damage with acceptable risk, our and others’ data might indicate that applying the cut-off point of the 95th percentile as the upper limit of normality seems to be a prudent approach to define normotension and controlled blood pressure as well as the point at which blood pressure should be managed with antihypertensive therapy [95, 104, 107].

Finally, further larger studies capable of generating robust statistical data, i.e., knowledge, are required to investigate whether the repeated use of ABPM significantly improves the physician’s decision to initiate or intensify antihypertensive therapy and to determine if this practice translates into important clinical benefits for the pediatric kidney transplant population.

## Key summary points

1. Ambulatory blood pressure monitoring is more accurate than blood pressure readings taken in the clinical setting to estimate an individual’s true mean blood pressure.
2. Several international professional groups have ascertained the utility of ABPM in the workup of pediatric hypertension, including post-renal transplant hypertension.
3. ABPM has the ability to identify patients with white-coat and masked hypertension and when performed at regular intervals, offers guidance for additional titration of antihypertensive medication as well as to confirm controlled hypertension.
4. Nocturnal hypertension and decreased blood pressure fall during sleep are well-described conditions after pediatric renal transplantation that can only be detected by ABPM.

## Multiple-choice questions (answers are provided following the reference list)

1. All statements below regarding the use of ABPM are true except one:
  - ABPM is inferior compared to blood pressure measurements taken in the clinical setting for improving a subject’s risk stratification.
  - ABPM identifies patients with white-coat hypertension.
  - ABPM identifies patients with masked hypertension.
  - ABPM provides a better estimate of a subject’s true mean blood pressure than blood pressure measurements taken in the clinical setting.
2. What is the best method of measuring blood pressure for the diagnosis and treatment monitoring of hypertension in renal transplant recipients?
  - Home blood pressure measurements.
  - ABPM.
  - Blood pressure measurements taken in the clinical setting.
  - All of them are alike.
3. What should be done in a recipient with hypertension that is diagnosed in the clinical setting and who has verified hypertensive-related organ damage?
  - Confirm hypertension by ABPM.
  - Start antihypertensive treatment without delay and monitoring treatment with ABPM.
  - After confirming hypertension by means of ABPM, treatment monitor should only rely upon blood pressure measurements taken in the clinical setting.
  - Answers a) and c) are correct.
4. In treated hypertensive renal transplant recipients with ambulatory controlled blood pressure:
  - The annual loss of renal allograft function is expected to be similar to normotensive recipients.
  - In hypertensive recipients with initially diagnosed left ventricular hypertrophy it is most likely to observe a regression of left ventricular mass under successfully long-term controlled blood pressure.
  - Uncontrolled hypertension has been shown to be associated with increased carotid intima-media thickness.
  - All answers are correct.
